# A Deployable LPWAN Platform for Low-Cost and Energy-Constrained IoT Applications

**DOI:** 10.3390/s19030585

**Published:** 2019-01-30

**Authors:** Bart Thoen, Gilles Callebaut, Guus Leenders, Stijn Wielandt

**Affiliations:** KU Leuven, ESAT-DRAMCO, Ghent Technology Campus, 9000 Ghent, Belgium; gilles.callebaut@kuleuven.be (G.C.); guus.leenders@kuleuven.be (G.L.); stijn.wielandt@kuleuven.be (S.W.)

**Keywords:** LPWAN, IoT, low power, sensor node, prototyping platform, Arduino, LoRaWAN

## Abstract

Many commercial platforms for fast prototyping have gained support for lpwan technologies. However, these solutions do not meet the low-cost and low-power requirements for a large-scale distribution of battery-powered sensor nodes. This paper presents the design, realization and validation of an open-source lpwan versatile platform. Energy and cost are considered key constraints for this hardware design. A power-efficient LoRa radio interface is implemented by hosting MAC functionality on the application microcontroller, eliminating the need for a modem. In the system architecture, power and cost savings are obtained by omitting and controlling lossy power circuitry. The resulting platform allows entry-level prototyping, while featuring an ultra-low sleep power of 25.2 μW. This makes lpwan sensor applications accessible in domains that would otherwise require custom hardware development. The proposed design is validated by an illustrative but functional example of sensor nodes deployed in the field.

## 1. Introduction

Numerous applications [1,2] require Internet of Things (IoT) devices to be operational for several years without manual intervention, i.e., deploy-and-forget. A vertical IoT application which is currently receiving a lot of attention is tracking or monitoring of commodities, such as the health of livestock. In case of scaling to a large number of end-devices, the overall expenditure of these systems often needs to be considered. As demonstrated in previous work [3], the cost of the employed devices and technologies is a key performance indicator.

The introduction of LPWANs has augmented the development of energy-efficient battery-powered IoT sensor nodes. A multitude of technologies is currently available, e.g., Long Range Wide Area Network (LoRaWAN) [4] NB-IoT [5], LTE-M [6], Wi-Fi HaLow (IEEE 802.11ah) and White-Fi (IEEE 802.11af). While LPWAN-enabled Wi-Fi standards exist, they did not experience the same traction as LoRaWAN or Sigfox. This is mainly due to the abandonment of Wi-Fi HaLow by chip manufacturers and the challenges White-Fi faces as a result of using TV white-space [7]. Cellular operators and Sigfox primarily focus on building IoT infrastructure, while the radio chips are designed by other vendors. These networks operate in a subscription-based manner. In contrast, Semtech, the founding company of Long Range (LoRa), develops the radio chips while the LoRaWAN open standard is developed and promoted by the LoRa Alliance. In LoRaWAN everyone can deploy their own network. Consequently, an end-user has three options to connect their device to a network, i.e., subscription-based, crowd-sourced, or private networks. In crowd-sourced networks, such as The Things Network, devices, servers, and gateways can be added to the network to extend the network. In addition, and in contrast to alternatives, the LoRaWAN MAC stack is publicly available [8], yielding numerous research opportunities.

### 1.1. Fast Prototyping and Deployment LoRaWAN Boards

The high accessibility (e.g., crowd-sourced networks) and flexibility (e.g., publicly available MAC stack) of LoRaWAN constitute to the adoption of this technology in a wide range of development platforms; for makers and educational or academic use. For instance, Reda et al. [9] uses LoRaWAN to provide meteorological information to rural farmers of developing countries. Equivalently, implementations of LoRaWAN nodes by means of integrating low-cost and open-source development platforms are presented in [10,11,12,13,14]. Several LoRaWAN development boards are already available, such as The Things Uno, an Arduino-based development platform provided by The Things Network to access their crowd-sourced network. Other Arduino-based platforms include, the MKR WAN 1300 developed and distributed by Arduino and the Seeeduino LoRaWAN hosting Grove connectors for the rapid assembly of sensors and actuators. In the remainder of the article these platforms are compared with the proposed entry-level LPWAN platform and serve as a baseline for comparison with our platform. Table 1 contains a summary of the relevant specifications of considered each platform, combined with those of the proposed solution. The previously described Arduino platforms focus on fast development rather than power efficiency. In addition to the Arduino-compatible boards, an open-source hardware-software platform for research in LPWANs is presented in [15], i.e., KRATOS. The KRATOS platform uses custom firmware based on ContikiOS and addresses the need for a low-power hardware design using a commercially available lora radio. This development board targets end-users requiring an operating system, resulting in a more expensive platform with a higher entry-level. In addition, we expect that the platform will be misused due to availability of an operating system, i.e., applications will be created which normally do not require an OS yielding a more expensive design and a higher power consumption. Notably, several other development boards are readily available, such as the B-L072Z-LRWAN1 discovery kit of ST. The platform supports the Mbed environment to facilitate developing application for IoT based on ARM microcontrollers.

### 1.2. Improving Arduino-Based Platforms

**Figure 1:**
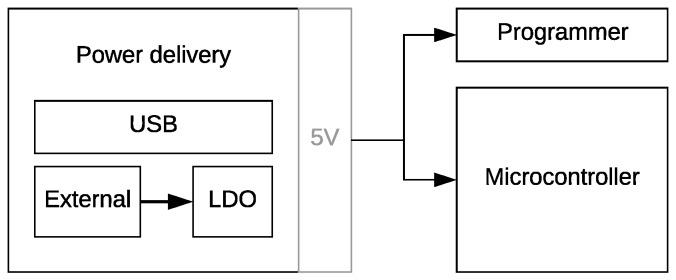
Arduino Uno power flow: power delivery either through USB or an external 12 V power supply, converted to a 5 V power rail powering both the always-on programmer and microcontroller.

Since the arrival of the Arduino Uno, fast prototyping and development platforms have gained popularity in e.g., educational settings, maker communities, research environments, and small-scale applications. Numerous libraries have been developed, by a vibrant community, to control a multitude of sensors, making Arduino easily accessible for people with little experience in embedded system design. The hardware design and the development tools are open source and free to use. As a result, variants on these boards have been developed, each with their own specific additions (e.g., wireless connectivity, an ARM processor, and a GPS receiver). Most of these boards are designed to remain compatible with the classic Arduino Uno layout. This implies, except for the classic pin header layout, a barrel jack DC power connector for external power bricks up to 12 V and a USB-connector to program and power the device from a USB port. While facilitating fast prototyping, ultra-low-power operation is usually not considered, as depicted in Figure 1. The USB programming circuitry and the use of a voltage regulator with a large quiescent current yield a higher power consumption than strictly required. As can be observed from Figure 1, the output of the Low-Dropout regulator (LDO) will act as a parasitic load and the on-board programming interface will draw unnecessary current when the board is powered by an external 5 V power supply. The combination of these effects contributes to a less energy-efficient design. The current Arduino-compatible boards primarily focus on lowering the barrier for creating IoT nodes. However, the solution proposed in this paper enhances these designs by employing low-power techniques to increase battery lifetime, while remaining consistent to the classic Arduino layout and microprocessor.

The main contribution of the presented work is the development of an open-source [16] low-cost lpwan platform for energy-constrained applications. The applied techniques to improve the energy efficiency of such systems is presented.

This paper is organized as follows. First, a brief overview of the LoRaWAN communication link is discussed, followed by a thorough description of the wireless sensor node design in Section 3. Afterwards, the characteristics of the complete system are elaborated and compared to commercially available solutions in Section 4. Section 5 demonstrates the applicability of the proposed solution by means of an example application, finalized by the conclusion.

## 2. LoRaWAN Communication Link Theory

LoRaWAN operates in license-exempt radio bands where transmissions are regulated. To comply with the regulations, LoRaWAN employs a duty cycle limit [8], constraining the number of transmissions per day. These limits range from 0.1% to 10% depending on the band and the region. Frequency Shift Keying (FSK) is selected for short range communication, while LoRa is specifically designed to transmit over larger distances. The coverage extension of the LoRa modulation is achieved by encoding symbols by means of a Chirp Spread Spectrum (CSS) technique. A symbol is spread over a fixed bandwidth using chirps. A LoRa symbol consists out of $2^{SF}$ chirps. Therefore, the symbol duration is based on the Spreading Factor (SF) and the bandwidth and is defined as:

(1) $$T_{\mathrm{sym}}=\frac{2^{SF}}{BW}$$

Thus, the data rate is inversely proportional to $2^{\mathrm{SF}}$. Consequently, decrementing the spreading factor results in doubling the data rate, while the coverage is extended when employing higher spreading factors due to the additional gain resulting from the spread spectrum technique. Therefore, a trade-off between energy consumption and range can be made dynamically by using different spreading factors. LoRaWAN employs Adaptive Data Rate (ADR) to optimize the power consumption depending on the radio propagation characteristics of the wireless channel. LoRaWAN specified different orthogonal spreading factors [17] ranging from 7 to 12. lora packets encoded with different spreading factors can be demodulated concurrently. The ability to make an energy versus range trade-off and the usage of orthogonal spreading factors in LoRaWAN results in a robust and long-range communication link for IoT devices.

### 2.1. Uplink-Centric MAC Design

Through a simple MAC scheme LoRaWAN, nodes can transmit messages in a low-power fashion. This scheme operates in a device-induced manner. When a node has an uplink message it wakes up and transmits this message. Hereafter, the node opens two receive windows where the gateway has the opportunity to send downlink messages to that node.

### 2.2. Accessing the Network

Prior to transmitting application data, nodes need to be personalized and activated. More specifically, to participate in a LoRaWAN network, security keys, and configuration parameters are required to be known at both the node and the back-end. Two joining procedures are defined to exchange this information, i.e., Activation By Personalisation (ABP) and Over The Air Activated (OTAA). In case of the former, the node is pre-registered on the network by programming this information on the node. A more secure option is OTAA where the keys are exchanged over the air. This approach allows to update the security keys without human intervention, i.e., re-programming the node.

## 3. Wireless Sensor Node Design

**Figure 2:**
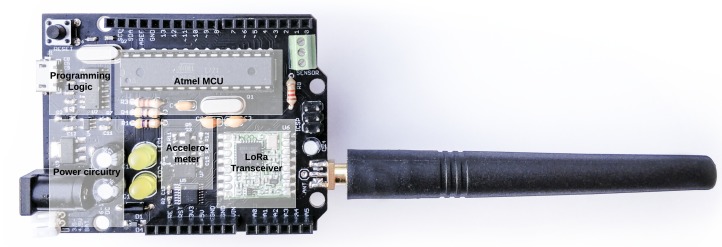
Picture of the proposed solution, including dipole antenna.

To facilitate generic prototyping needs, while still being low power, low cost, and low entry, a new platform was developed. This platform, depicted in Figure 2, features the classic Arduino-compatible Microcontroller Unit (MCU) (i.e., ATmega328P), a redesigned efficient power delivery circuit, a wireless LoRaWAN-enabled transceiver, and access to other peripherals. The remainder of this section is structured as follows. First, an overview of the node architecture is presented including the power circuit and the wireless transceiver. Hereafter, the energy provision is considered, where the battery-type selection and other related rationales are discussed in more detail.

### 3.1. Node Architecture

**Figure 3:**
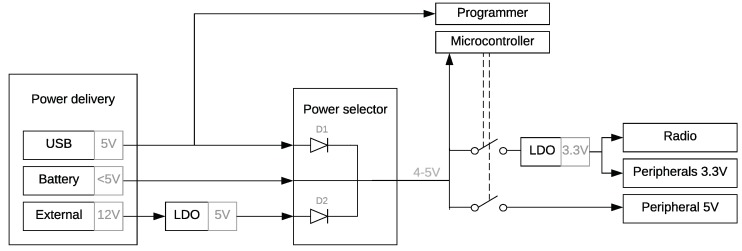
Power flow of the proposed solution: power can be delivered through the USB port, an external battery, or an external 12 V supply. The most efficient power rail is selected to power the MCU and other peripherals. The programmer is only activated when the USB port is active. The on-board radio and other peripherals can be turned off in sleep mode.

The sensor node, depicted in Figure 3, is designed as a versatile IoT platform, while enabling a low-power system design and maintaining a high community support. The architecture comprises the power selector, MCU and accompanying programmer, radio module, and other peripherals.

#### 3.1.1. Power Selector

In contrast to the aforementioned platforms, the optimal power delivery technology is selected by means of a power selector, thereby drastically reducing the quiescent power consumption. The module can be powered by the on-board USB port, an external battery (typically 3 AA batteries in series) or an external 12 V power supply. The programmer is only powered when the module is powered by the USB interface, because the interface is only necessary when the board is plugged into a computer. Powering down the interface results in significant power savings and is ensured by diode D1 (Figure 3) which prevents voltage from being applied to the programmer when no USB voltage is present. An additional diode, i.e., diode 2, is employed to reduce current draw by the LDO when powered by the USB or battery, otherwise the LDO would behave as a parasitic load. By introducing both diodes, the power rails can be combined to provide 5 V to other components. In this design, we opted for the low-cost On Semiconductor 1N4149 diodes with low leakage current (25 nA); further reducing the quiescent power of the module. Equivalently, a low-cost LDO is chosen, i.e., Texas Instruments LM1117, to convert the 12 V power rail to 5 V. An LDO is preferred over a switched-mode converter, as it fits the low-cost objective. Battery protection is implemented in the mechanical design of the node, making it impossible to connect an external power supply and a battery at the same time.

#### 3.1.2. Arduino-Vompatible MCU

By using an Arduino software compatible MCU, the combination of the low-entry programming language and the community of Arduino is exploited to facilitate prototyping and developing IoT devices. The Microchip ATmega328P MCU was chosen over other commonly used Arduino-compatible controllers, e.g., Microchip ATmega32u4 and Microchip SAM D21, for complete Arduino compatibility while maintaining low cost and low power. If the MCU is put in sleep mode, power consumption can be as low as 0.3 μW [18]. The MCU can be programmed through an on-board and low-cost programmer (WCH CH340). By still featuring an on-board programming interface, the proposed platform can be used out-of-the-box, without requiring any external hardware. The programming interface is fully compatible with the Arduino Integrated Development Environment (IDE). Hence, all Arduino libraries, functions, and other software can be used with the proposed low-power development board.

#### 3.1.3. LoRa Transceiver

LoRaWAN modems employ a dedicated MCU to provide a convenient interface (e.g., through AT-commands) as well as hosting the LoRaWAN MAC functionality. This additional MCU contributes to a higher cost and power consumption. Moving this functionality to the host MCU (ATmega32u4), hence, reduces the cost and power consumption. Furthermore, hosting the MAC-layer on the host MCU allows us to advance the stack by using available hardware accelerations and sleep modes. For instance, the host MCU can be put in sleep between the transmit and receive windows. Notably, the attainable improvements depend on the used host MCU.

For the proposed solution, a Semtech SX1276-based lora transceiver is selected. It features three distinct power amplifiers. Two unregulated power-efficient amplifiers covering the LoRaWAN-defined [17] low and high frequency bands. A high-power Power Amplifier (PA) can be used to deliver up to 20 dBm in contrast to the efficient amplifiers supporting only a maximum of 14 dBm. Numerous transceiver chip vendors favor using the high-power PA on the Semtech SX127x series due to its extensive capabilities. More specifically, the high-power PA covers all frequency bands of interest (e.g., 433 MHz and 868 MHz) and supports transmission at 20 dBm. However, current LoRaWAN network implementations only allow end-devices to transmit in bands of maximum 14 dBm. To give an example, the current consumption during a transmission when using the high-power pa is 120 mA compared to 24 mA with the more efficient HF-pa. For this reason, we opted to select the unregulated HF-pa for high-power efficiency delivering up to 14 dBm. Opposed to the popular HopeRF RFM95 and RF Solution RF-LORA-868, used by the KRATOS board, and other low-cost equivalent transceivers, we optimized the power efficiency by opting for the HF-pa amplifier. The LoRa transceiver is preferred over a LoRaWAN modem for being low-cost, low-power, and versatile, e.g., €4 for the HopeRF RFM95 compared to €11 for the popular Microchip RN2483 LoRa modem. In addition, the Semtech SX1276 supports both LoRaWAN [19] and LoRa communication [20]. Via the latter, LoRa modulation can be used to set up point-to-point communication [21] or an own MAC design can be employed.

The module features three types of antenna connections, i.e., u.fl, SMA, and a wire connection. This allows some flexibility in selecting the most optimal antenna-type in an application-agnostic manner. A PCB antenna is not provided due to size constraints.

#### 3.1.4. Peripherals

The wireless transceiver operates between 1.8 V and 3.7 V. To maintain Arduino compatibility, a 3.3 V output is needed. To obtain a cost-effective and stable voltage supply for peripherals, the presented solution uses a 3.3 V LDO voltage regulator (Texas Instruments LP2985). The power can be cut off when no radio or other 3.3 V peripheral is needed, since the LDO is controlled by the host MCU. This reduces the sleep current to an absolute minimum. The Serial Peripheral Interface (SPI) interface, required for communicating with the tranceiver, is converted to 3.3 V by a low-cost level shifter (74HC4050). An electronic switch for 5 V peripherals is included, enabling the user to power 5 V peripherals when needed. The board also includes an accelerometer (Analog Devices ADXL345) and can be used as an on-board testing peripheral. User friendly Arduino integration is maintained, because numerous libraries for this accelerometer have been developed by the Arduino community. Furthermore, the ADXL345 provides an interrupt functionality allowing other components to be put in sleep mode or turned off when no change in orientation is detected.

### 3.2. Energy Provision

To provide energy to the wireless sensor node, a suitable power source is required. AA alkaline primary batteries are a suitable candidate, because they are readily available at a low price point [22]. Compared to other primary batteries the alkaline battery has the highest capacity to price ratio [23]. They have a nominal voltage of 1.5 V, a cut-off voltage of 1 V and after 5 years after manufacturing 70% of the initial capacity remains [24]. With a nominal voltage of 1.5 V most devices require multiple batteries connected in series. Three AA batteries are, in most cases, capable of powering devices designed for 5 V. For 3.3 V devices generally two batteries suffice. A lower voltage eliminates the need for an LDO, saving valuable energy. However, the supply voltage will drop as the battery discharges. Therefore, the circuit and used components will determine the lower voltage limit, fixing the usable amount of energy that can be extracted from the battery. Table 2 gives the battery voltage levels corresponding to the remaining battery charge. A load of 330 mW is considered to overestimate the capacity loss when transmitting (110 mW).

Comparing the maximum supply voltage of the different boards from Table 1, the amount of AA batteries can be selected. Except for the MKR WAN 1300 with a supply voltage of 3.3 V all boards can operate on three AA batteries in series. The MKR WAN 1300 will be powered by two AA batteries in series. To define the battery capacity available to each platform, the drop-out voltage of each platform was measured and summarized in Table 3. The MKR WAN 1300 has the lowest expected usable energy, because it only operates on two batteries. The proposed solution stops working at voltages below 3.3 V, resulting in an available capacity of 21 kJ. With an available energy of ± 30 kJ The Things Uno and Seeeduino LoRaWAN perform better, because they can operate at voltages below 3.3 V.

## 4. Assessment of the Energy Efficiency and Expected Lifetime

The energy efficiency of the proposed solution is evaluated by conducting power measurements on the considered boards (Table 1). The measurements were performed at room temperature.

**Figure 4:**
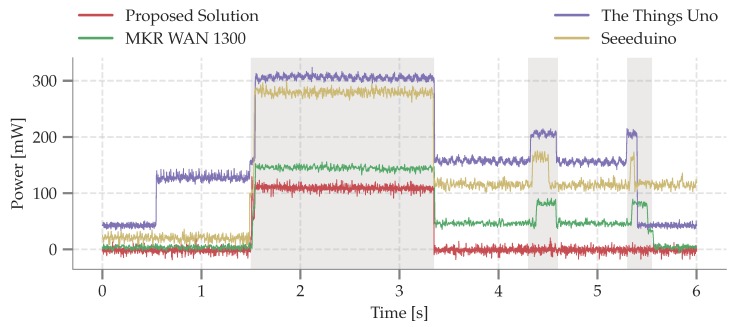
Power consumption of the considered platforms when transmitting a packet of 20 Bytes at SF12 with a transmit power of 14 dBm. The platform wakes up, transmits a packet, and opens two receive windows.

**Figure 5:**
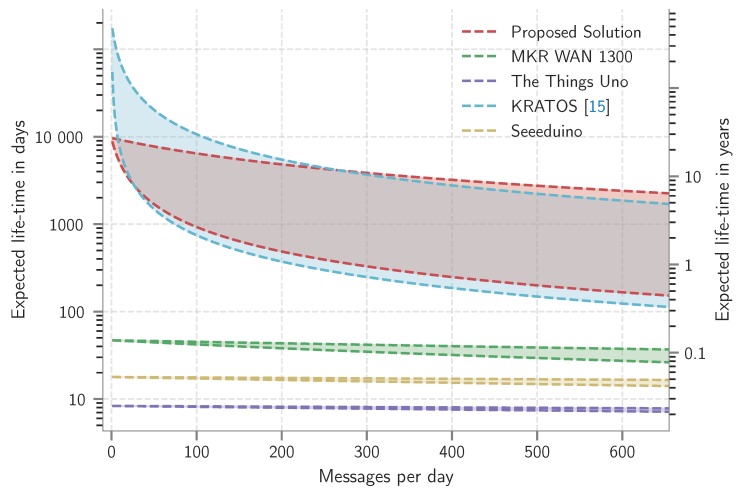
Expected operation time in terms of messages per day for spreading factors 7 (upper-bound) and 12 (lower-bound) with a 20 Byte payload. The maximum of 655 messages per day corresponds to the 1% duty cycle for transmitting a 20-byte message with SF12.

First, the power consumption in different power states is measured and summarized in Table 4 and visualized in Figure 4. Secondly, the minimum operating voltage, i.e., the drop-out voltage, is determined for each platform. Based on the drop-out-voltage, the available battery capacity, and the power profile the expected operation lifetime is estimated. The result of this study is depicted in Figure 5.

### 4.1. Power Profile of Different Power States

The power profile of each considered platform is measured by transmitting a packet of 20 Bytes at 14 dBm. A 20 Byte packet was used conform the packet length of the example application elaborated in Section 5. Before the transmission is initiated, each board is in its sleep state. The MCU wakes and configures the transceiver to transmit a packet with SF7 or SF12 respectively. After transmitting the packet, the board returns to sleep state. In the sleep state, the MCU is put in sleep mode and peripherals are shut down; if supported. For instance, some boards do not support turning off LEDs, contributing to additional measured sleep power. Since low-level library adjustments and hardware tweaks for energy optimization are not considered a low-entry approach, the comparison of the considered platforms does not include these modifications. Because our solution has control over the LoRaWAN MAC stack, the energy efficiency is improved by omitting the receive windows. Consequently, the node is directly put in sleep mode after the transmission.

The transitions between different power states is illustrated in Figure 4. The proposed solution clearly outperforms the The Things Uno and Seeeduino LoRaWAN. The Things Uno is the least power-efficient due to a large setup time and the high sleep power (42.7 mW). Hence, The Things Uno is not suited for battery-powered operation. While performing slightly better than the previous board, a similar conclusion can be made when observing the power profile of Seeeduino LoRaWAN. Moreover, the Seeeduino LoRaWAN library takes up to 6 s to put the LoRaWAN modem in sleep. The long initialization phase is due to configuring the RisingHF RHF76 transceiver. The MKR WAN 1300 performs similar to the proposed solution; with a low sleep power of 3.7 μW and a slightly higher power consumption in transmit mode (+13% with respect to our solution). Hence, the main difference in power consumption between the MKR WAN 1300 and the proposed platform is the presence of the receive window. As observed in [26], retransmissions are predominately a result of overloaded gateways opposed to lost messages. Hence, in our setup no retransmissions are requested thereby lowering the power consumption as a consequence of halting the radio before opening the receive windows.

### 4.2. Expected Lifetime

The expected lifetime is determined based on the aforementioned power measurements and the battery datasheet information. Since room temperature operation is considered, realistic battery lifetimes will most likely deteriorate in adverse weather conditions. Figure 5 illustrates the expected lifetime of the platforms in terms of the number of transmissions per day for a message of 20 Bytes at 14 dBm. Each graph is upper-bounded by the expected lifetime when sending with SF7, while the lower-bound depicts transmitting with SF12. The starting point, i.e., one packet per day, is determined by the quiescent power of the platforms. As the number of messages per day increases, the expected lifetime is determined by the power related to the lora transmissions. As can be observed in Figure 5, KRATOS and the proposed solution have an expected lifetime of over 10 years opposed to the other platforms. The high quiescent power of the MKR WAN 1300, Seeeduino LoRaWAN and The Things Uno contributes to a low lifespan of several days. The expected lifetime of the KRATOS platform drops more quickly compared to the proposed platform with increased number of transmitted messages per day. The difference in power consumption between the KRATOS and the proposed solution is a result of the combined effect of using the more efficient pa and omitting the receive slots. Since an Alkaline battery loses its charge over time, 30% after 5 years, this graph is an idealistic representation of the expected lifetime. In realistic situations the self-discharge rate should be taken in to account.

## 5. Example Application

**Figure 6:**
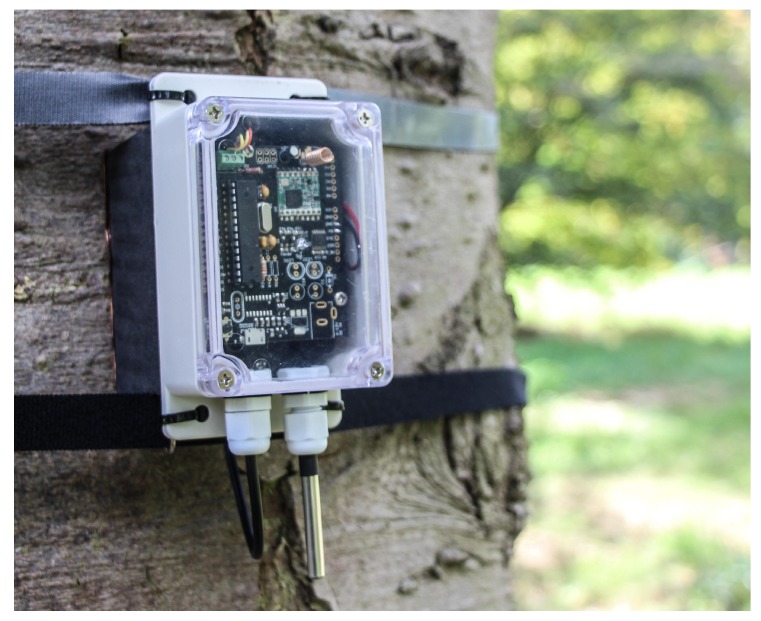
Application: the proposed platform is employed as a sensor for monitoring the health of trees.

The presented platform is currently being used in a project called IoTree. In this project, a LoRaWAN network is deployed to monitor the health of trees (Figure 6). Prior to deploying the nodes, a range test is performed determining the maximum achievable distance between a tree and the gateway. Our field measurements demonstrate coverage up to 1 km with antennas at only 1.5 m height in an urban scenario. In Line-of-Sight (LoS) this coverage is extended to 4 km, meeting the long range requirements. To ensure a battery lifetime of up to one year some low-power strategies are adopted in the application design of the IoTree node. These strategies are not limited to this project, but could be used in other scenarios. The IoTree nodes measure the temperature of the tree trunk combined with the outside temperature using two DS18B20 one-wire temperature sensors. From these two temperature measurements, the juice flow in the tree can be derived. The on-board accelerometer is employed to monitor the inclination of the tree, monitoring large structural deformations of the tree, e.g., when the tree is blown down during a storm or if the tree starts to sink. The battery voltage is measured using the internal 1.1 V reference of the ATmega328P. The measured data is transmitted over the radio in byte efficient format (CayenneLPP), resulting in a data-frame of 20 bytes. To save power, ABP is employed to conveniently wake-up the transceiver without requiring re-joining. In case of OTAA, the LoRaWAN stack needs to support storing the generated keys in non-volatile memory; which is not implemented in the available open-source code. Removing the join procedure also eliminates the need for the receive windows, reducing the time the radio is activated. Each 15 min a measurement is performed, consisting of the readout of the sensors (190 ms @ 65.7 mW), transmitting the data packet of 20 bytes at SF12 resulting in an expected lifetime of 2.5 years for the proposed solution. However, the derived lifetime neglects the self-discharge of the used alkaline batteries. When running the example application on the KRATOS platform, we expect a similar lifetime of 2 years. The other benchmark platforms only last a couple of days; with 8 days for The Things Uno, 17 days for Seeeduino LoRaWAN and 43 days for the MKR WAN. This conclusion confirms the observations made in Figure 5. However, in contrast to Figure 5, in the example application the lifetime is reduced by powering the additional sensors.

## 6. Conclusions

The increased interest in LPWANs has accelerated the development of different IoT platforms for fast prototyping and deployment. A myriad of these platforms has adopted LoRaWAN for LPWAN connectivity. Since the arrival of the Arduino Uno, fast prototyping and development platforms have gained popularity in e.g., educational settings, maker communities, research environments, and small-scale applications. These platforms mainly focus on fast prototyping but neglect the low-power aspect, resulting in a high sleep current. Consequently, these boards are not suitable for testing and deploying sensor nodes that require operating times of multiple years. The solution proposed in this paper addresses this low-power aspect, while remaining fully Arduino Uno compatible. Depending on the sf, the proposed solution can operate for 20 years on SF7 or 3.8 years on SF12 and this for 96 messages, or a message every 15 min, of 20 bytes. Compared to other Arduino-compatible development platforms such as The Things Uno, MKR WAN 1300 and Seeeduino LoRaWAN this is a significant improvement, since they do not manage to last longer than 0.2 years in the most efficient configuration. The proposed platform is open-source and currently put to the test in a research project for monitoring the health of trees, called IoTree.

## Figures and Tables

**Table 1:** Overview of the benchmarked sensor boards.

	Proposed Solution	MKR WAN 1300	The Things Uno	Seeeduino LoRaWAN	KRATOS [15]
Host MCU	ATmega328p	SAMD21	ATmega32u4	ATSAMD21	TI MSP430
Modem	Semtech SX1276 chip	Murata CMWX1	Microchip RN2483	RisingHF RHF76	Semtech SX1276 chip
Software Environment	Arduino	Arduino	Arduino	Arduino	ContikiOS
Supply voltage	5 V	3.3 V	5 V	5 V	5 V
Price	<€30	€33	€55	€47	<€100
Antenna Connector	SMA/u.fl/wire-antenna	u.fl	u.fl	u.fl.	SMA
Built-in Antenna	-	-	PCB antenna	Wire-antenna	-

**Table 2:** Cell voltage for a 330 mW load in terms of remaining capacity of an AA Alkaline battery (10800 J) [25].

**Capacity [%]**	100	90	80	70	60	50	40	30	20	10	0
**330** m W **load [V]**	1.49	1.35	1.27	1.20	1.16	1.12	1.10	1.08	1.04	0.98	0.62

**Table 3:** Expected usable energy considered platforms.

	Proposed Solution	MKR WAN	The Things Uno	Seeeduino LoRaWAN
**Battery configuration**	3AA	2AA	3AA	3AA
**Drop-out Voltage [V]**	3.27	2.17	2.25	2.54
**Drop-out Voltage per cell [V]**	1.09	1.08	0.75	0.85
**Untapped capacity [%]**	35	30	5	6
**Expected Usable Energy [J]**	21,060	15,120	30,780	30,456

**Table 4:** Power consumption of the considered platforms.

	Proposed Solution	MKR WAN	The Things Uno	Seeeduino LoRaWAN
**Power [mW]**			
Sleep Power	0.025	3.726	42.75	19.75
Transmit Power SF7	91.35	141.5	225.9	288.9
Transmit Power SF12	111.15	144.80	300.92	279.9
**Energy [mJ]**			
Radio Pre-processing	0.36	2.28	117.85	766.87
RX window SF7	0	106.65	325.83	171.75
RX window SF12	0	113.91	335.05	172.98

## Abbreviations

The following abbreviations are used in this manuscript:

ABP	Activation By Personalization
ADR	Adaptive Data Rate
CSS	Chirp Spread Spectrum
FSK	Frequency Shift Keying
IDE	Integrated Development Environment
IoT	Internet of Things
LDO	Low-Dropout regulator
LoRa	Long-Range
LoRaWAN	Long-Range Wide Area Network
LoS	Line-of-Sight
LPWAN	Low Power Wide Area Network
MAC	Medium Access Control
MCU	Microcontroller Unit
OTAA	Over the Air Activated
PA	Power Amplifier
SF	Spreading Factor
SPI	Serial Peripheral Interface
